# Comprehensive analysis of alternative splicing and transcriptome diversity in apple using long-read sequencing

**DOI:** 10.3389/fpls.2026.1819201

**Published:** 2026-05-11

**Authors:** Chenyang Hu, Xin Huang, Shuzhen Luo, Hong Zhang, Wenzhou Chen, Yuhua Niu, Chengnan Xu, Xiangqian Zhang, Yongxin Zhao, Ruigang Wu, Jirong Zhao

**Affiliations:** 1 Shaanxi Key Laboratory of Research and Utilization of Resource Plants on the Loess Plateau, College of Life Sciences, Yan’an University, Yan’an, Shaanxi, China; 2 Institute of Biotechnology, Beijing Academy of Agriculture and Forestry Sciences, Beijing, China; 3 Engineering Research Center of Microbial Resources Development and Green Recycling, University of Shaanxi Province, Yan’an, Shaanxi, China; 4 Research and Development Centre of Ecological and Sustainable application of Microbial Industry of the Loess Plateau in Shaanxi Province, Yan’an, Shaanxi, China; 5 College of Chemistry and Chemical Engineering, Shaanxi University of Science and Technology, Xi’an, Shaanxi, China; 6 College of Landscape and Ecological Engineering, Hebei University of Engineering, Handan, Hebei, China

**Keywords:** alternative splicing atlas, apple, full-length transcriptome, tissue-specific, transcriptome diversity

## Abstract

**Introduction:**

Alternative splicing (AS) is a major source of transcriptome diversity in plants, but short-read RNA sequencing (RNA-seq) has limited power for resolving full-length transcripts and complex AS patterns, particularly in perennial crops.

**Methods:**

We integrated Oxford Nanopore Technology (ONT) long-read cDNA sequencing with Illumina RNA-seq to characterize the full-length transcriptome across seven apple (*Malus domestica*) tissues, including vegetative organs and three fruit developmental stages. Gene- and transcript-level expression were quantified, and AS variation was assessed using percent spliced in (PSI) and differential splicing analyses.

**Results:**

We identified 56,809 genes and 100,911 transcript isoforms and constructed an AS atlas comprising 29,842 events across seven AS types, involving 17,659 genes and 73,067 transcript isoforms. These analyses revealed extensive tissue-associated transcriptional and post-transcriptional regulation, including large sets of tissue-specific genes, transcript isoforms, and AS events, as well as widespread differential splicing between tissue pairs.

**Discussion:**

Together, our results show that integrating ONT long reads for isoform discovery with Illumina short reads for quantitative support improves apple transcriptome annotation and enables systematic characterization of tissue-resolved transcript and AS variation. The resulting full-length transcriptome, unified transcript annotation, and AS atlas provide a valuable resource for studies of post-transcriptional regulation, trait-associated transcript variation, and genetic improvement in apple.

## 1 Introduction

Alternative splicing (AS) is a pervasive process in eukaryotes in which a single pre-mRNA can generate multiple transcript isoforms (Pan et al., 2008; Reddy et al., 2013; Xu et al., 2024). Genome-wide analyses have shown that more than 60% of plant intron-containing genes undergo AS (Reddy et al., 2013; Niu et al., 2024). As a major post-transcriptional regulatory mechanism, AS substantially increases transcript and protein diversity (Nilsen and Graveley, 2010; Elkon et al., 2013; Laloum et al., 2018; Zhao et al., 2019; Du et al., 2024).

Advances in high-throughput sequencing have greatly expanded transcriptome-wide studies of gene regulation (Filichkin et al., 2010; Thatcher et al., 2016; Zhang et al., 2016; Jiang et al., 2017; Tu et al., 2022; Tyagi et al., 2022). Early transcriptome analyses relied mainly on short-read next-generation sequencing (NGS). Although NGS offers high throughput, low per-base error rates, and cost-effective sequencing, its short read length limits the resolution of transcript structures. In particular, short reads may not map uniquely in repetitive or highly similar genomic regions, and full-length isoforms often have to be reconstructed from fragmented reads, which constrains accurate characterization of complete transcript structures and complex AS events (Zhang et al., 2020).

Third-generation long-read sequencing has overcome many of these limitations by producing reads that can extend to tens of kilobases and frequently span full-length transcripts (Wang et al., 2021; Espinosa et al., 2023; Espinosa et al., 2024). Long-read platforms, including Oxford Nanopore Technologies (ONT) and PacBio, enable direct characterization of transcript isoforms and AS patterns with reduced dependence on transcript reconstruction from short reads (Koren et al., 2012; Zhao et al., 2019; Espinosa et al., 2024). Full-length transcript coverage improves the detection and interpretation of complex AS events, including exon skipping, intron retention, and alternative 5′/3′ splice-site usage, and enhances the identification of low-abundance or structurally complex isoforms that are difficult to resolve using short-read sequencing alone (Hou et al., 2021; Hu et al., 2022; Li et al., 2022a). Nevertheless, long-read transcriptomics is often complemented by short-read RNA-seq, which provides higher per-base accuracy and greater sequencing depth for robust expression quantification. Accordingly, integrated long-read and short-read strategies are increasingly being used to combine comprehensive isoform discovery with reliable transcriptome quantification.

Here, we integrated ONT full-length transcriptome sequencing with Illumina RNA-seq to characterize the transcriptome landscape across seven apple tissues. At the genome-wide level, we identified 56,809 genes, 100,911 transcript isoforms, and 29,842 AS events. We further assessed differential gene expression, transcript expression, and differential alternative splicing across tissues. These results provide a useful resource for improving apple transcriptome annotation and for investigating post-transcriptional regulation in apple.

## 2 Materials and methods

### 2.1 Plant materials and sampling

Samples were collected from the National Germplasm Repository of Apple at the Institute of Pomology, Chinese Academy of Agricultural Sciences, Xingcheng, Liaoning, China (120.72°E, 40.62°N). Ten-year-old ‘Golden Delicious’ trees with moderate vigor were used as the experimental material. Roots, stems, leaves, flowers, and fruits were sampled, and fruits were collected at 45, 95, and 145 days after blooming (DAB), corresponding to the juvenile, expanding, and mature stages, respectively. One sample per tissue was collected for ONT full-length transcriptome sequencing, and three biological replicates per tissue were collected for Illumina RNA-seq. All samples were immediately frozen in liquid nitrogen after collection and stored at −80 °C until use.

### 2.2 ONT and Illumina RNA-seq library preparation and sequencing

ONT libraries were prepared according to the manufacturer’s standard protocol. For full-length transcriptome sequencing, 1 μg of total RNA from one sample per tissue was used to construct cDNA libraries with the cDNA-PCR Sequencing Kit (SQK-PCS109; ONT, Oxford, UK). Libraries were generated using a sequencing library pre-amplification kit containing PCR adapters at the ends of first-strand cDNA. PCR amplification was performed for 20 cycles with LongAmp Taq, and ONT adapters were then ligated to the PCR products using T4 DNA ligase. The amplified products were purified with Agencourt XP beads according to the ONT protocol. Final cDNA libraries were loaded onto FLO-MIN109 flow cells and sequenced on a MinION Mk1B sequencer.

For Illumina RNA-seq, approximately 2 μg of RNA per sample was used for library construction. Libraries were sequenced on a HiSeq X-ten platform (Illumina, Inc., San Diego, CA, USA) using a 150-bp paired-end strategy, generating 21 libraries in total (seven tissues × three biological replicates).

### 2.3 ONT RNA-seq data processing

Raw nanopore data in fast5 format were base-called and converted to fastq format using Guppy (v2.2.3) in MinKNOW (v2.2) (Wick et al., 2019). Quality control was performed with NanoPlot (v1.42.0) (De Coster et al., 2018), and reads shorter than 500 bp or with an average quality score < 7 were removed. Full-length non-chimeric reads were identified using Pychopper (v2.7.10). The filtered reads were aligned to the reference genome using Minimap2 (v2.27) (Li, 2018) with the parameters -ax splice -uf -k14 -G 1000000, and BAM files were generated for each tissue.

### 2.4 Illumina RNA-seq data processing

Raw Illumina reads were filtered using fastp (v0.23.0) (Chen et al., 2018) with the parameters -q 20 -l 50 -n 0.05 -5 -M 15 -w 4 to obtain clean reads and calculate Q20, Q30, and GC content. Clean reads from each biological replicate were aligned independently to the reference genome (GDDH13_1-1_formatted.fasta.gz) (Daccord et al., 2017) using HISAT2 (v2.2.1) (Kim et al., 2019), and BAM files were generated with SAMtools (v1.17) (Li et al., 2009). For transcript model construction, the three Illumina replicate alignments from the same tissue were combined at the tissue level and integrated with the corresponding ONT long-read alignment. For expression quantification and differential expression analyses, however, the three biological replicates were retained and analyzed independently.

### 2.5 Transcript model construction and functional annotation

For each tissue, StringTie (v2.2.3) (Pertea et al., 2015) was run in --mix mode to integrate the tissue-level Illumina short-read alignment with the corresponding ONT long-read alignment, using the reference annotation supplied with the -G option to generate tissue-level transcript models. The resulting GTF files from the seven tissues were merged into a unified nonredundant transcript annotation (All.gtf). This annotation was compared with the reference gene annotation (gene_models_20170612.gff3.gz) using gffcompare (v0.12.6) to classify known and novel genes and transcripts. The final unified annotation was used as the common reference for downstream quantification and AS-event generation.

Coding sequences were predicted from the unified transcript set using TransDecoder (v5.7.1). Functional annotation of the predicted protein sequences was performed with eggNOG-mapper (emapper.py v2.1.13; November 2024 annotation framework), and the resulting assignments were used to retrieve annotations from GO (v1.2; accessed 2024-11-05) (Ashburner et al., 2000), KEGG (v112.0; accessed 2024-11-05) (Kanehisa and Goto, 2000), COG (v24.0; accessed 2024-11-05) (Tatusov et al., 2000), Swiss-Prot (v2024-11; accessed 2024-11-05) (the UniProt Consortium, 2017), and Pfam (v37.0; accessed 2024-11-05) (Mistry et al., 2021).

### 2.6 Identification and quantification of genes, transcript isoforms, and AS events

Gene- and transcript-level analyses were performed using the unified transcript annotation (All.gtf), whereas the sequencing data used at each step differed according to analytical purpose. ONT long reads were used primarily for transcript structure discovery, annotation refinement, and AS-event definition, whereas Illumina short reads were used for quantitative analyses across the 21 biological replicate libraries.

Gene-level read count matrices were generated from Illumina short-read alignments against All.gtf using StringTie (v2.2.3) and the Python script prepDE.py (Pertea et al., 2015). Differentially expressed genes (DEGs) were identified using DESeq2 (v1.46.0) (Love et al., 2014) with a fold change ≥ 2 and a false discovery rate (FDR) < 0.05. Transcript sequences were extracted from All.gtf using gffread (v0.12.7), and transcript abundance was quantified from Illumina short reads using Salmon (v1.9.0) (Patro et al., 2017) based on the All.gtf-derived transcript reference. Differentially expressed transcripts (DETs) were identified using Ballgown (v2.38.0) in R (v4.4) under Bioconductor (v3.20), with a fold change ≥ 2 and an FDR < 0.05.

AS events were generated from the unified transcript annotation using the generateEvents function in SUPPA2 (v2.2) (Trincado et al., 2018). PSI values and differential alternative splicing (DAS) were then assessed using transcript abundance estimates derived from the short-read data. DAS events were considered significant at *P* < 0.05 with an absolute change in PSI (|ΔPSI|) ≥ 0.05. GO and KEGG enrichment analyses for genes, transcripts, and genes associated with at least one AS event were performed using clusterProfiler (v4.14.6) (Yu et al., 2012), with q < 0.05 as the significance threshold.

In this study, TSGs, TSTs, and TSAS events were defined operationally according to the current sequencing depth, expression threshold, and PSI estimability criteria. These categories should therefore be interpreted as tissue-restricted features identified under the present analytical conditions rather than as absolutely tissue-exclusive biological features.

## 3 Results

### 3.1 Overview of ONT and Illumina RNA-seq data

To profile the apple transcriptome across seven tissues, total RNA was collected from roots, stems, leaves, flowers, immature fruits (45 DAB), expanding fruits (95 DAB), and mature fruits (145 DAB). Seven tissue-specific ONT cDNA libraries (one library per tissue) and 21 Illumina RNA-seq libraries (three biological replicates per tissue) were constructed. ONT sequencing generated 5.52–7.29 Gb of clean bases and 4,435,713–5,807,414 clean reads per tissue, with mean read lengths of 1,244.3–1,346.3 bp, median read lengths of 1,056–1,188 bp, read length N50 values of 1,393–1,511 bp, and mean quality scores of Q8 (**Supplementary Table 1**). After full-length read identification, 3,587,082–4,693,774 full-length reads were obtained per tissue, representing 85.84%–87.86% of the reads, and 97.16%–99.37% of ONT reads mapped to the reference genome (**Supplementary Table 2**). For Illumina RNA-seq, the 21 libraries yielded 23,182,824–38,758,571 clean reads and 6.93–11.58 Gb of clean bases per library, with Q30 scores of 93.59%–95.42% and GC contents of 47.09%–48.28% (**Supplementary Table 3**). The overall mapping rate of Illumina reads ranged from 88.73% to 94.95%, and the uniquely mapped read percentage ranged from 76.80% to 87.84% across libraries (**Supplementary Table 3**). Together, these results indicate that both the ONT and Illumina datasets were of sufficient quality to support transcript structure characterization and downstream quantitative analyses.

### 3.2 ONT RNA-seq data analysis

Analysis of the full-length transcriptome data identified 56,809 genes, including 31,189 known genes and 25,620 novel genes (**Supplementary Table 4**). Among these, 15,612 genes had two or more transcript isoforms (**Supplementary Table 4**). In total, 100,911 transcripts were detected, comprising 33,031 known transcripts, 31,570 novel-in-catalog (NIC) transcripts, and 36,310 novel-not-in-catalog (NNIC) transcripts (**Supplementary Table 5**). Across tissues, the number of detected genes ranged from 44,664 to 51,001, including 26,510–29,855 known genes and 18,154–21,146 novel genes, whereas the number of genes with two or more transcript isoforms ranged from 14,134 to 15,145. The number of detected transcripts per tissue ranged from 76,097 to 86,318, including 26,082–30,072 known transcripts, 25,022–27,150 NIC transcripts, and 24,993–29,097 NNIC transcripts (**Supplementary Tables 4**, **5**). These results indicate that long-read-supported transcriptome reconstruction substantially expanded transcript and isoform resolution in apple.

### 3.3 Analysis of gene expression levels and identification of tissue-specific genes

**Figure 1:**
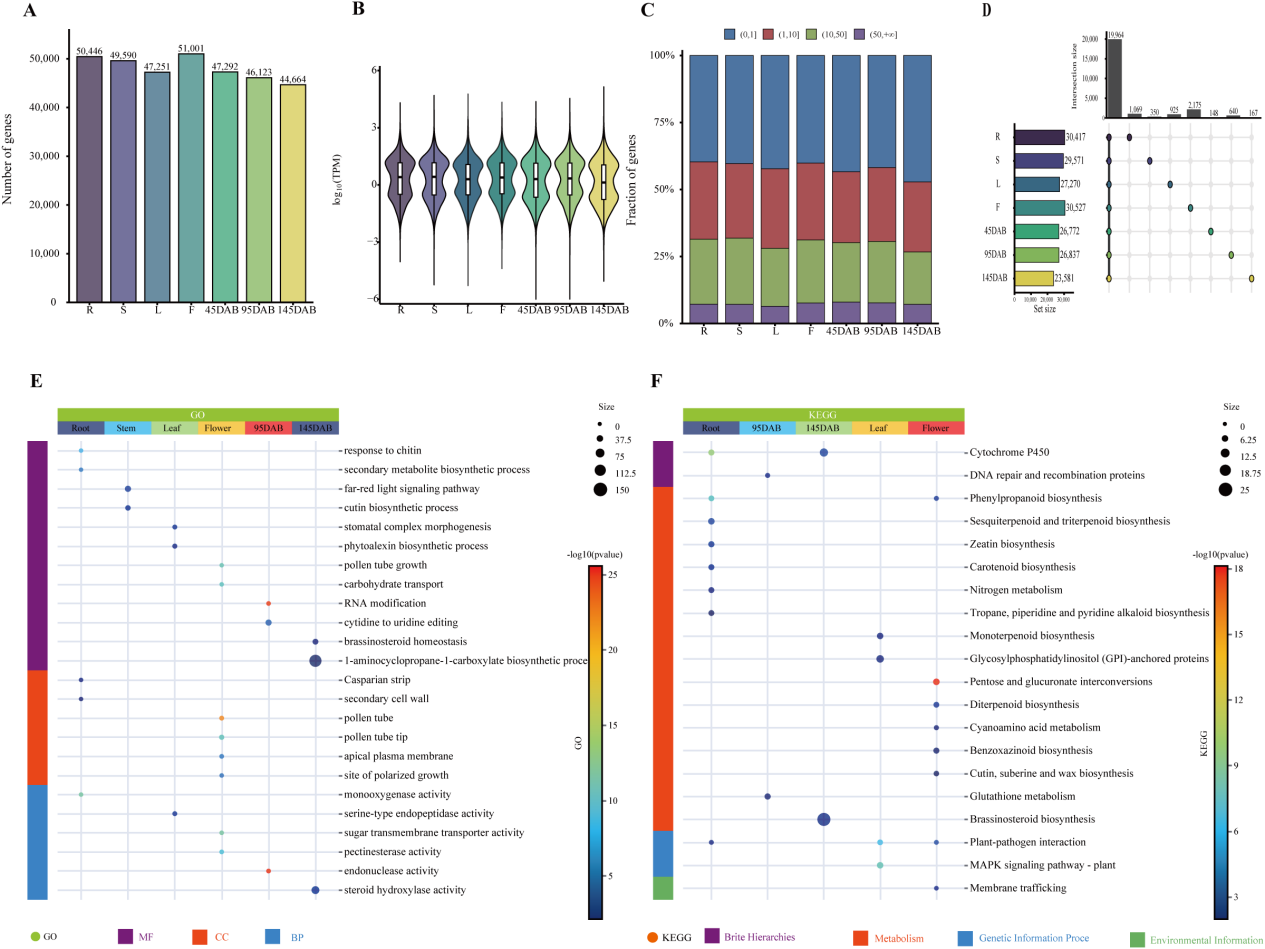
Corrected gene-level expression landscape and tissue-specific gene analysis across seven apple tissues. **(A)** Number of expressed genes (TPM > 0) in each tissue. **(B)** Distribution of gene expression levels in each tissue, shown as log10 (TPM). **(C)** Proportional distribution of genes across four expression classes: (0,1], (1,10], (10,50], and (50, +∞] TPM. **(D)** Compact UpSet plot showing the sizes and major intersections of gene sets across seven tissues; single-set counts correspond to tissue-specific genes under the current analytical criteria. **(E)** Go, Gene Ontology enrichment analysis of tissue-specific gene-associated sets across seven tissues. **(F)** KEGG, Kyoto Encyclopedia of Genes and Genomes enrichment analysis of tissue-specific gene-associated sets across seven tissues. Tissue abbreviations are as follows: R, root; S, stem; L, leaf; F, flower; immature fruit (45 DAB), expanding fruit (95 DAB), and mature fruit (145 DAB).

Of the 56,809 genes quantified using the unified transcript annotation (All.gtf), 55,906 were expressed in at least one tissue (mean TPM > 0; **Supplementary Table 6**). The number of expressed genes per tissue ranged from 44,664 to 51,001 (**Figure 1A**), and the mean TPM among expressed genes ranged from 19.52 to 22.32 (**Figure 1B**). Classification by expression abundance further showed that lowly expressed genes (0 < TPM ≤ 1) constituted the largest fraction across tissues, followed by moderately expressed genes (1 < TPM ≤ 10), whereas highly expressed (10 < TPM ≤ 50) and very highly expressed genes (TPM > 50) accounted for progressively smaller proportions (**Figure 1C**). Tissue-restricted genes ranged from 148 to 2,175 across tissues (**Figure 1D**; **Supplementary Table 6**). Their functional profiles were strongly tissue dependent, broadly partitioning into defense and secondary metabolism in roots, epidermal and cuticle-related development in leaves, reproductive growth in flowers, and stage-dependent regulatory functions during fruit development (**Figures 1E, F**). At the GO level, these signals were represented by enrichment of chitin response and secondary metabolite biosynthetic processes in roots, cutin biosynthesis and stomatal complex morphogenesis in leaves, pollen tube growth and polarized growth in flowers, RNA modification and nucleic-acid-related processes at 95 DAB, and brassinosteroid- or steroid-associated processes at 145 DAB (**Figure 1E**). At the KEGG level, these tissue-restricted gene sets were further associated with cytochrome P450- and phenylpropanoid-related pathways in roots, DNA repair/recombination and nitrogen metabolism at 95 DAB, cutin/suberine/wax and glutathione metabolism at 145 DAB, and signaling-related pathways such as plant–pathogen interaction and MAPK signaling in leaves and flowers (**Figure 1F**), while the broader TSG, DEG, and TSDEG related gene sets further emphasized stimulus response, photosynthesis/chloroplast functions, carbohydrate metabolism, secondary metabolism, and DNA repair/recombination processes (**Supplementary Tables 7**, **8**).

Across the 21 pairwise tissue comparisons, the number of differentially expressed genes (DEGs) ranged from 8,333 in the root vs. stem comparison to 20,487 in the leaf vs. 145 DAB fruit comparison. Tissue-specific differentially expressed genes (TSDEGs), defined here as significant DEG subsets unique to a single pairwise comparison, ranged from 21 to 437 and were most abundant in the leaf vs. 45 DAB fruit comparison. Consistent with the TSG patterns, these differential gene sets were mainly enriched in processes related to external stimulus response, plastid- and chloroplast-associated functions, photosynthesis, carbohydrate metabolism, secondary metabolism, and DNA repair/recombination (**Supplementary Tables 7**, **8**).

### 3.4 Analysis of transcript expression levels and identification of tissue-specific transcripts

**Figure 2:**
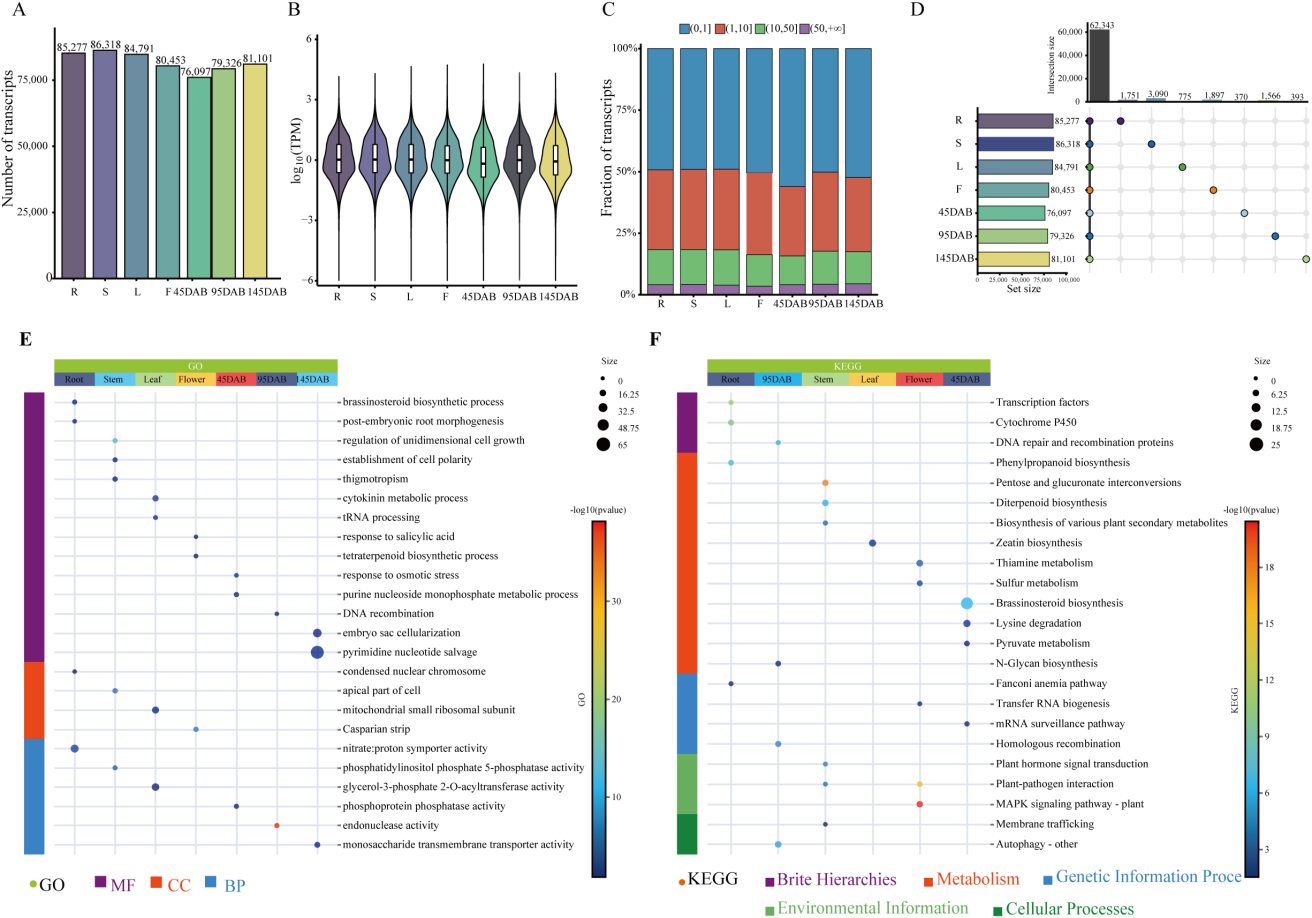
Analysis of transcript expression and tissue-specific transcript analysis in seven apple tissues. **(A)** Number of expressed transcripts in each tissue (TPM>0); **(B)** Average transcript expression level in each tissue [log10(TPM)]; **(C)** Distribution of transcript expression levels; **(D)** Number of tissue-specific transcripts; **(E)** GO, Gene Ontology analysis of tissue-specific transcripts. **(F)** KEGG, Kyoto Encyclopedia of Genes and Genomes analysis of tissue-specific transcripts. Here are the seven tissue names and their corresponding abbreviations: R, root; S, stem; L, leaf; F, flower; juvenile stages fruit (45 DAB), expansion stages fruit (95 DAB), and maturity stages fruit (145 DAB).

Among the 100,911 annotated transcripts, 97,644 were expressed in at least one tissue (mean TPM > 0; **Supplementary Table 9**). The number of expressed transcripts per tissue ranged from 76,097 to 86,318 (**Figure 2A**), and the mean TPM among expressed transcripts ranged from 11.59 to 13.14 (**Figure 2B**). Classification by expression abundance further showed that lowly expressed transcripts (0 < TPM ≤ 1) constituted the largest fraction across tissues, followed by moderately expressed transcripts (1 < TPM ≤ 10), whereas highly expressed (10 < TPM ≤ 50) and very highly expressed transcripts (TPM > 50) accounted for progressively smaller proportions (**Figure 2C**). Tissue-restricted transcripts ranged from 370 to 3,090 across tissues (**Figure 2D**; **Supplementary Table 9**). Their functional profiles were also strongly tissue dependent, but at the transcript level the dominant signals were more closely associated with developmental polarity and root-related growth, stress and hormone responses, RNA/nucleotide metabolism, and membrane- or transporter-associated functions (**Figures 2E, F**). At the GO level, these patterns were represented by enrichment of post-embryonic root morphogenesis, cell polarity and unidimensional growth, salicylic acid and osmotic stress responses, tRNA processing, DNA recombination, nucleotide salvage, and transporter-related activities across different tissue-restricted transcript sets (**Figure 2E**). At the KEGG level, these transcript-restricted sets were further associated with transcription factors and cytochrome P450 in roots, pentose and glucuronate interconversions in stems, plant–pathogen interaction and MAPK signaling in flowers, brassinosteroid biosynthesis in 45 DAB fruits, and transfer RNA biogenesis, mRNA surveillance, homologous recombination, and membrane trafficking in 95 DAB fruits (**Figure 2F**), while the broader TST, DET, and TSDET related transcript-equivalent gene sets extended these trends to transcript-associated programs linked to development, RNA processing, signaling, transport, and genome maintenance (**Supplementary Table 10**, **11**).

The number of differentially expressed transcripts (DETs) was highest between flowers and 95 DAB fruits (n = 12,450), whereas several closely related contrasts contained only 1–4 DETs. Tissue-specific differentially expressed transcripts (TSDETs), defined here as significant DET subsets unique to a single pairwise comparison, ranged from 2 to 5,534, with the highest count also observed between flowers and 95 DAB fruits. Consistent with the TST patterns, these transcript-level differential sets mainly emphasized transcript-associated functions related to development, RNA processing, signaling, transport, and genome maintenance (**Supplementary Table 10**, **11**).

### 3.5 AS profiling and tissue-specific AS events

**Figure 3:**
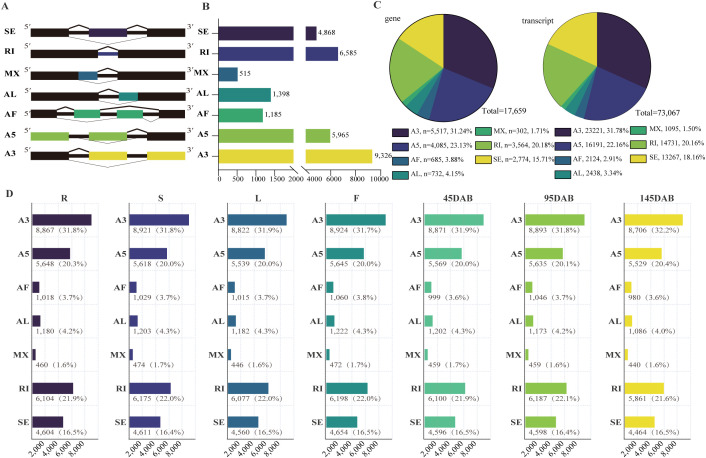
Analysis of alternative splicing and tissue-specific AS events in seven apple tissues. **(A)** Identification of AS types [SE, skipped exon; RI, retained intron; MX, mutually exclusive exon; AF, alternative first exon; AL, alternative last exon; A5, alternative 5′ splice site; and A3, alternative 3′ splice site]; **(B)** Quantitative statistics of AS events in all tissues; **(C)** Number of genes and transcripts involved in each AS event; **(D)** Quantitative statistics of each AS event in each tissue. Here are the seven tissue names and their corresponding abbreviations: R, root; S, stem; L, leaf; F, flower; juvenile stages fruit (45 DAB), expansion stages fruit (95 DAB), and maturity stages fruit (145 DAB).

Using SUPPA2, we identified seven classes of AS events, including alternative 3′ splice sites (A3), alternative 5′ splice sites (A5), alternative first exons (AF), alternative last exons (AL), mutually exclusive exons (MX), retained introns (RI), and skipped exons (SE) (**Figure 3A**). In total, 29,842 AS events were detected, with A3 representing the most prevalent class (9,326; 31.2%) and MX the least frequent class (515; 1.7%) (**Figure 3B**). These events involved 17,659 genes and 73,067 AS-associated transcript isoforms (**Figure 3C**; **Supplementary Table 12**). Notably, the overall AS composition was highly similar across tissues, with A3 consistently remaining the dominant class, MX the rarest class, and A5, RI, and SE together accounting for a substantial proportion of the AS landscape in all seven tissues (**Figure 3D**), indicating that the global repertoire of AS event types is broadly stable across tissues, whereas tissue specificity is more evident at the level of event usage and splicing regulation.

Consistent with this interpretation, the number of AS events with quantifiable PSI per tissue ranged from 27,678 to 28,832, and 2,366 tissue-specific AS (TSAS) events were identified overall, with 170–591 TSAS events detected per tissue and the largest TSAS set observed in roots (**Supplementary Table 12**). Functionally, these TSAS-associated gene sets further indicated clear tissue dependence, highlighting floral development and reproductive processes in flowers, stimulus-responsive functions in fruit tissues, and tissue-dependent enrichment of spliceosome-, transcription-, and translation-related pathways (**Supplementary Table 12**; **Supplementary Figures 2**, **3**).

A similarly strong tissue-dependent pattern was observed for DAS. Across the 21 pairwise tissue contrasts, 17,206 significant DAS event-comparison rows were detected, with the highest count in stem vs. 95 DAB fruit (n = 1,609) and the lowest in root vs. leaf (n = 414). These significant comparisons collapsed to 5,088 unique DAS events, and 3,668 tissue-specific DAS event–tissue rows were identified, with 95 DAB fruits contributing the largest set (n = 1,337); together, these results indicate that although the overall AS-event composition is broadly conserved, the regulation of local splice choices varies substantially among tissues, particularly during fruit development (**Supplementary Tables 13**-**15**).

### 3.6 Integrated candidate prioritization across gene, transcript, and AS layers

**Figure 4:**
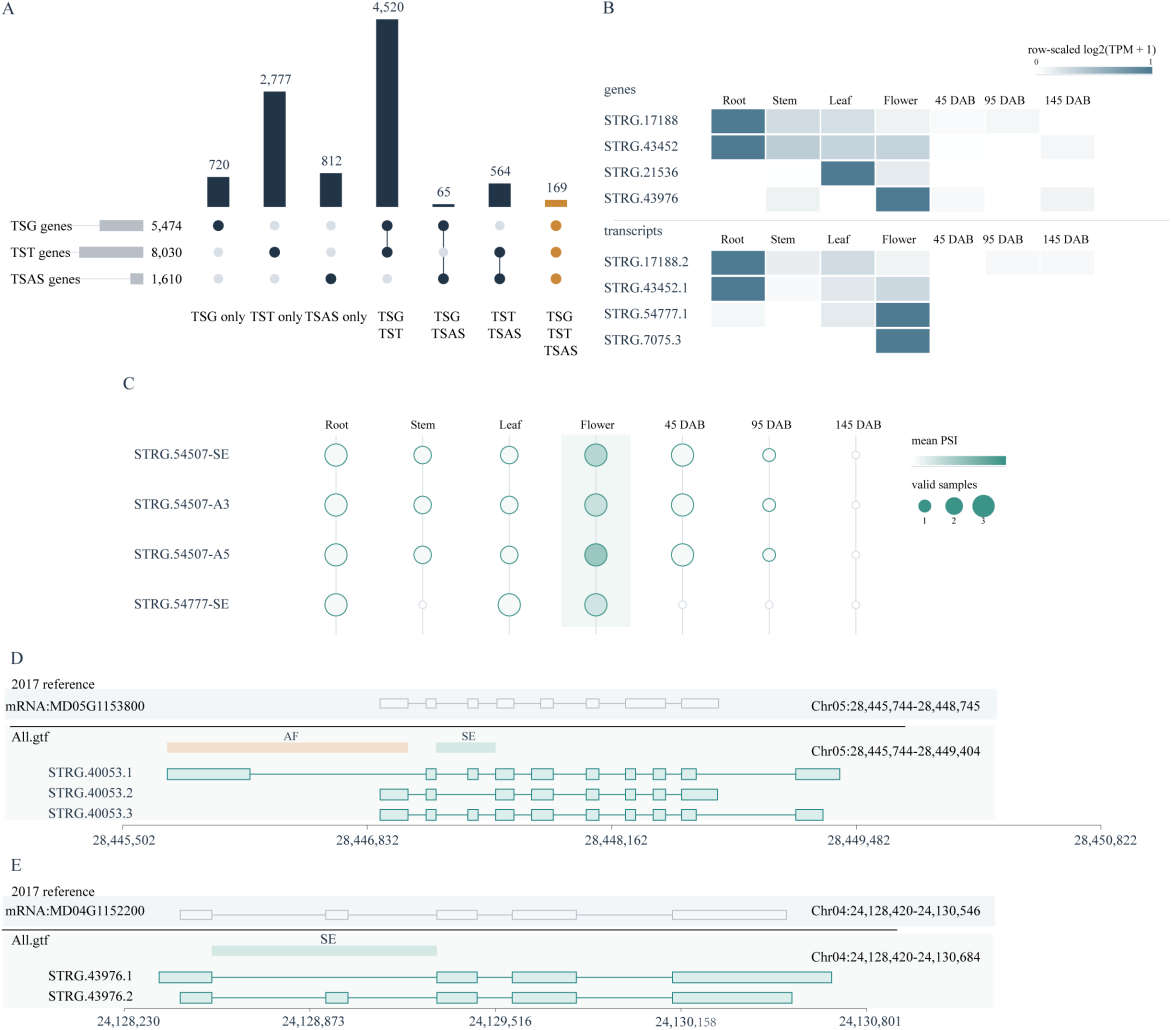
Integrated multilayer regulation and annotation refinement in apple. **(A)** UpSet summary of the direct overlap among genes supported by TSG, tissue-specific gene expression, genes associated with TST, tissue-specific transcripts; and genes associated with TSAS, tissue-specific AS events. The direct three-way overlap contains 169 genes and is distinct from the downstream prioritized candidate framework summarized in **Supplementary Table 16**. **(B)** Representative gene- and transcript-level heatmaps showing raw mean TPM values and row-scaled expression patterns across seven tissues. Gene-level examples include *STRG.17188*, *STRG.43452*, *STRG.21536*, and *STRG.43976*, and transcript-level examples include *STRG.17188.2*, *STRG.43452.1*, *STRG.54777.1*, and *STRG.7075.3*. **(C)** Bubble plot of representative AS events showing tissue-resolved PSI patterns. Bubble color indicates mean PSI, and bubble size indicates the number of valid samples used for PSI estimation in each tissue. **(D)** Annotation refinement at the *STRG.40053* locus relative to the 2017 reference annotation and the corrected All.gtf, showing long-read-supported transcript models and representative alternative first-exon and skipped-exon features. **(E)** Annotation refinement at the *STRG.43976* locus relative to the 2017 reference annotation and the corrected All.gtf, showing corrected isoform structure and an associated skipped-exon event.

To determine whether tissue-associated regulation was coordinated across multiple transcriptomic layers, we compared tissue-specific genes (TSGs), genes associated with tissue-specific transcripts (TST-associated genes), and genes associated with tissue-specific AS events (TSAS-associated genes). This comparison showed that most tissue-associated signals were layer-specific or shared by only two layers, whereas a smaller core set displayed coordinated evidence across all three layers. Specifically, among 5,474 TSGs, 8,030 TST-associated genes, and 1,610 TSAS-associated genes, 169 genes were shared by all three sets (**Figure 4A**), identifying a high-confidence core of loci simultaneously supported by gene-expression, transcript-usage, and tissue-specific splicing evidence.

Representative loci further illustrated how this multilayer coordination was manifested in specific tissues. At the gene level, *STRG.17188* and *STRG.43452* showed root-enriched expression, *STRG.21536* showed leaf-enriched expression, and *STRG.43976* showed flower-enriched expression (**Figure 4B**; **Supplementary Table 6**). At the transcript level, *STRG.17188.2* and *STRG.43452.1* were enriched in roots, whereas *STRG.54777.1* and *STRG.7075.3* showed flower-enriched transcript expression (**Figure 4B**; **Supplementary Table 9**). At the splicing level, three representative events from *STRG.54507* (SE, A3, and A5) and one SE event from *STRG.54777* showed flower-biased PSI patterns (**Figure 4C**; **Supplementary Table 12**). Together, these examples indicate that tissue-associated regulation in apple can be coordinated across gene expression, transcript isoform usage, and local splicing behavior, particularly in root- and flower-associated regulatory programs. At the annotation level, long-read-supported transcript models further refined the STRG.40053 and STRG.43976 loci, revealing alternative first-exon and skipped-exon features at STRG.40053 (**Figure 4D**) and a corrected isoform structure with an associated skipped-exon event at STRG.43976 (**Figure 4E**).

Beyond this direct three-way overlap, the broader downstream prioritization framework further organized multilayer-supported candidates into a 203-gene high-confidence set, a top 100 prioritized subset, an extended multilayer candidate set, and an all-gene integrated support summary (**Supplementary Table 16**). Thus, **Figure 4A** defines the most stringent overlap-based core, whereas **Supplementary Table 16** expands this signal into a structured resource for downstream candidate prioritization and functional follow-up.

## 4 Discussion

Short-read RNA-seq has greatly advanced plant transcriptomics, but accurate reconstruction of full-length isoforms remains challenging in species with structurally complex genomes and extensive transcript diversity. This issue is particularly relevant in apple, whose relatively recent whole-genome duplication increases sequence similarity among paralogous loci and complicates isoform-level inference from fragmented reads alone. In this context, the present study provides a tissue-resolved full-length transcriptome framework for cultivated apple by integrating ONT long reads with Illumina-based quantification across seven tissues. Rather than serving only as a technical advance, this dataset expands transcriptomic resolution in a biologically meaningful way, placing apple alongside other fruit crops in which long-read-supported transcriptomics has substantially improved isoform discovery and annotation, including citrus, kiwifruit, and wild apple (Tang et al., 2016; Liu et al., 2021; Hu et al., 2022).

A major outcome of this work is that the improved annotation is not limited to a larger transcript count, but also provides clearer structural interpretation at specific loci. In our dataset, the *STRG.40053* locus is resolved into three transcript isoforms with additional alternative first-exon and skipped-exon configurations, whereas *STRG.43976*, which was represented more simply in the 2017 reference annotation, is resolved into two transcript isoforms together with a skipped-exon event. These examples show that long-read-supported reconstruction can refine locus-level transcript architecture rather than merely add more transcript models, which is particularly valuable for perennial fruit crops in which trait-relevant loci may be regulated through subtle isoform differences rather than presence–absence alone.

Our AS results further indicate that the apple transcriptome combines global compositional stability with strong tissue-dependent local regulation. At the whole-transcriptome level, A3 was the dominant AS class and MX the rarest, and the overall class composition was broadly similar across tissues. This pattern is closer to the A3-enriched profile reported in citrus than to the RI-dominant patterns described in several herbaceous species, reinforcing the view that dominant AS classes can vary across lineages and biological contexts (Hu et al., 2022). At the same time, the TSAS and DAS analyses show that conserved global proportions do not imply uniform splicing behavior: local splice choices were strongly tissue dependent, with especially prominent variation across fruit developmental stages. This fruit-stage signal is biologically plausible because AS has been shown to shift dynamically during fleshy fruit development and ripening in kiwifruit, peach, papaya, melon, and cucumber, where developmental stage can reshape the balance among isoforms and their associated regulatory pathways (Tang et al., 2016; Yan et al., 2021). Our results therefore support a model in which the apple fruit transcriptome is regulated not only through gene-expression changes, but also through progressive reprogramming of transcript usage and local splice decisions during development.

This developmental interpretation is further strengthened by emerging apple and fruit-tree evidence that AS can directly influence agronomic traits rather than simply accompany them. In apple, alternative splicing of the *ALMT9/Ma1* transporter has recently been shown to modulate vacuolar malate transport and fruit acidity, demonstrating that isoform differences can have direct functional consequences for fruit quality (Li et al., 2024). In pear, AS variation has also been linked to domestication- and improvement-related fruit traits, including firmness, sugar/acid metabolism, and stone cell formation, supporting the idea that transcript-level regulation can contribute to phenotypic divergence in fruit trees (Li et al., 2022b). Against this background, the fruit-stage TSAS and DAS signals detected here should be regarded as potentially relevant to developmental- and quality-associated regulation in apple, even though functional validation remains necessary before any specific event can be assigned a causal role.

Finally, the multilayer analysis provides a practical bridge from atlas construction to candidate prioritization. Most tissue-associated signals remained layer-specific or were shared by only two layers, but a smaller direct three-way overlap of 169 genes defined a more stringent core supported simultaneously by tissue-restricted gene expression, transcript usage, and splicing evidence. This distinction between the direct overlap in **Figure 4A** and the broader prioritization framework in **Supplementary Table 16** should be maintained, because the latter is intentionally more inclusive and designed for downstream follow-up rather than strict overlap interpretation. Within this framework, representative loci such as *STRG.17188* and *STRG.43452* in roots, *STRG.21536* in leaves, *STRG.43976* and *STRG.54777* in flowers, and the flower-biased splicing events of *STRG.54507* and *STRG.54777* illustrate how tissue-associated regulation can emerge through coordinated changes across multiple transcriptomic layers. These examples are best viewed as high-confidence entry points for future functional work, especially in relation to root physiology, floral regulation, and fruit developmental biology, rather than as functionally confirmed regulators at the current stage.

## 5 Conclusion

ONT RNA-seq enabled a comprehensive characterization of the apple transcriptome across seven tissues. By integrating a unified transcript annotation with updated downstream analyses, we expanded apple gene and isoform annotation, defined tissue-resolved gene, transcript, and AS landscapes, and characterized differential expression and differential splicing across 21 tissue contrasts. The integrated multilayer analysis further identified a direct three-way overlap of 169 genes supported by tissue-specific gene expression, tissue-specific transcript usage, and tissue-specific AS evidence, while **Supplementary Table 16** provides a broader prioritized candidate framework for future functional studies.

## Data Availability

The raw ONT and Illumina sequencing data generated in this study have been deposited in the China National Center for Bioinformation under accession number PRJCA033992. The unified transcript annotation (All.gtf), functional annotation files, and supporting source data have been deposited in Zenodo at https://doi.org/10.5281/zenodo.19682911. Gene-, transcript-, and AS-related result tables are also provided in **Supplementary Tables 1**-**16**.

## Supplementary material

Supplementary Tables 1–16 Corrected final supplementary tables supporting the gene-, transcript-, and alternative splicing-related analyses in this study.

Supplementary Figures S1–S3 Corrected final supplementary figures supporting the functional enrichment and tissue-specific alternative splicing analyses in this study.
