# Supplementary material for: Comprehensive analysis of alternative splicing and transcriptome diversity in apple using long-read sequencing
Source: Front Plant Sci. 2026 May 11;17:1819201. doi: 10.3389/fpls.2026.1819201 (PMC13199297; doi:10.3389/fpls.2026.1819201)

Supplementary Fig. S3. KEGG enrichment analysis of genes associated with tissue-specific alternative splicing (TSAS) events across seven apple tissues.

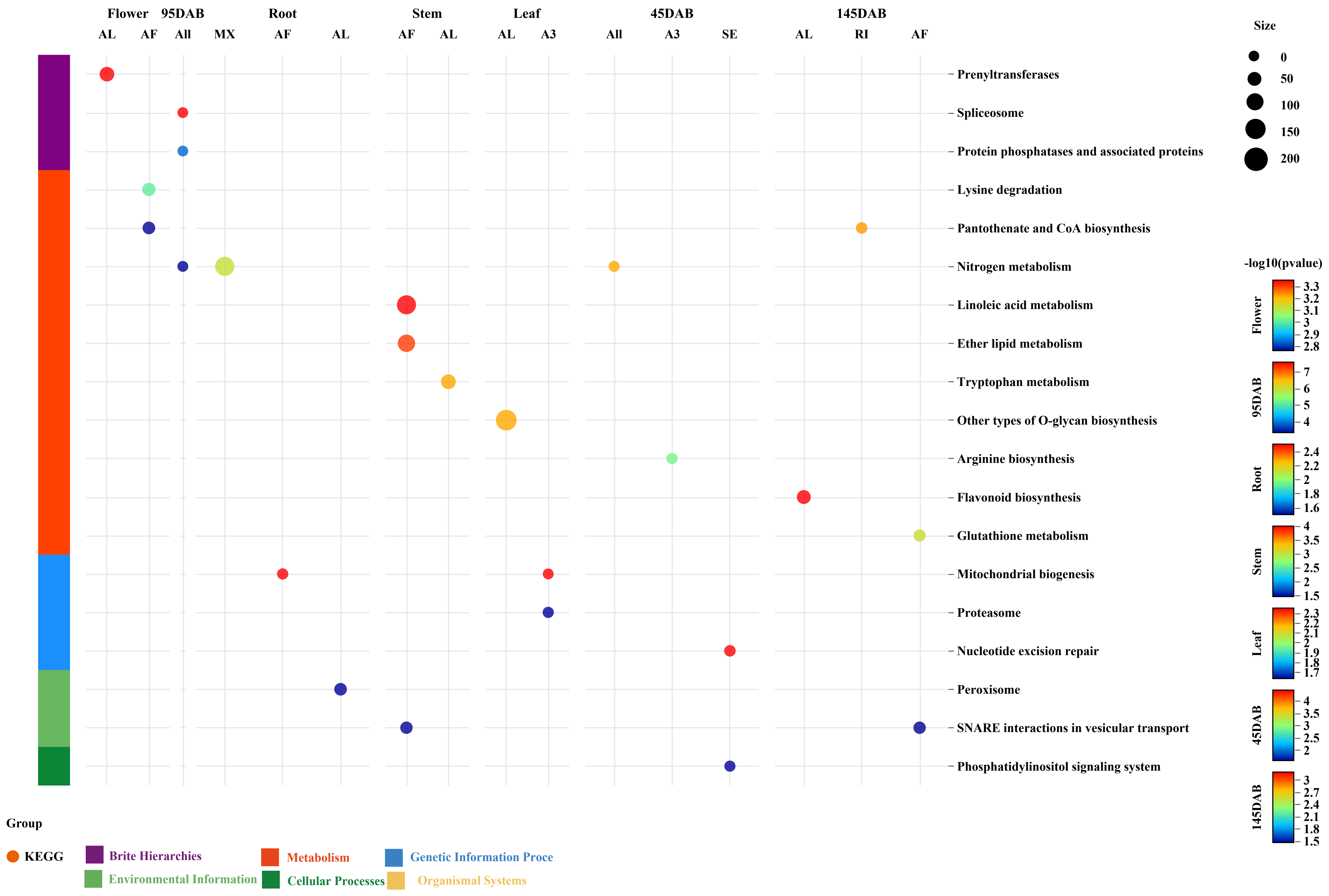

Supplement: Supplementary Figures S1–S3 — Corrected final supplementary figures supporting the functional enrichment and tissue-specific alternative splicing analyses in this study. [file DataSheet2.zip › Supplementary Fig. S3.pdf]
