# Supplementary material for: Comprehensive analysis of alternative splicing and transcriptome diversity in apple using long-read sequencing
Source: Front Plant Sci. 2026 May 11;17:1819201. doi: 10.3389/fpls.2026.1819201 (PMC13199297; doi:10.3389/fpls.2026.1819201)
Supplement: Supplementary Figures S1–S3 — Corrected final supplementary figures supporting the functional enrichment and tissue-specific alternative splicing analyses in this study. [file DataSheet2.zip › Supplementary Fig. S1.pdf]

Supplementary Fig. S1. Overview of the bioinformatics workflow used for transcriptome annotation refinement, gene- and transcript-level quantification, alternative splicing identification, and downstream integrative analyses in apple.

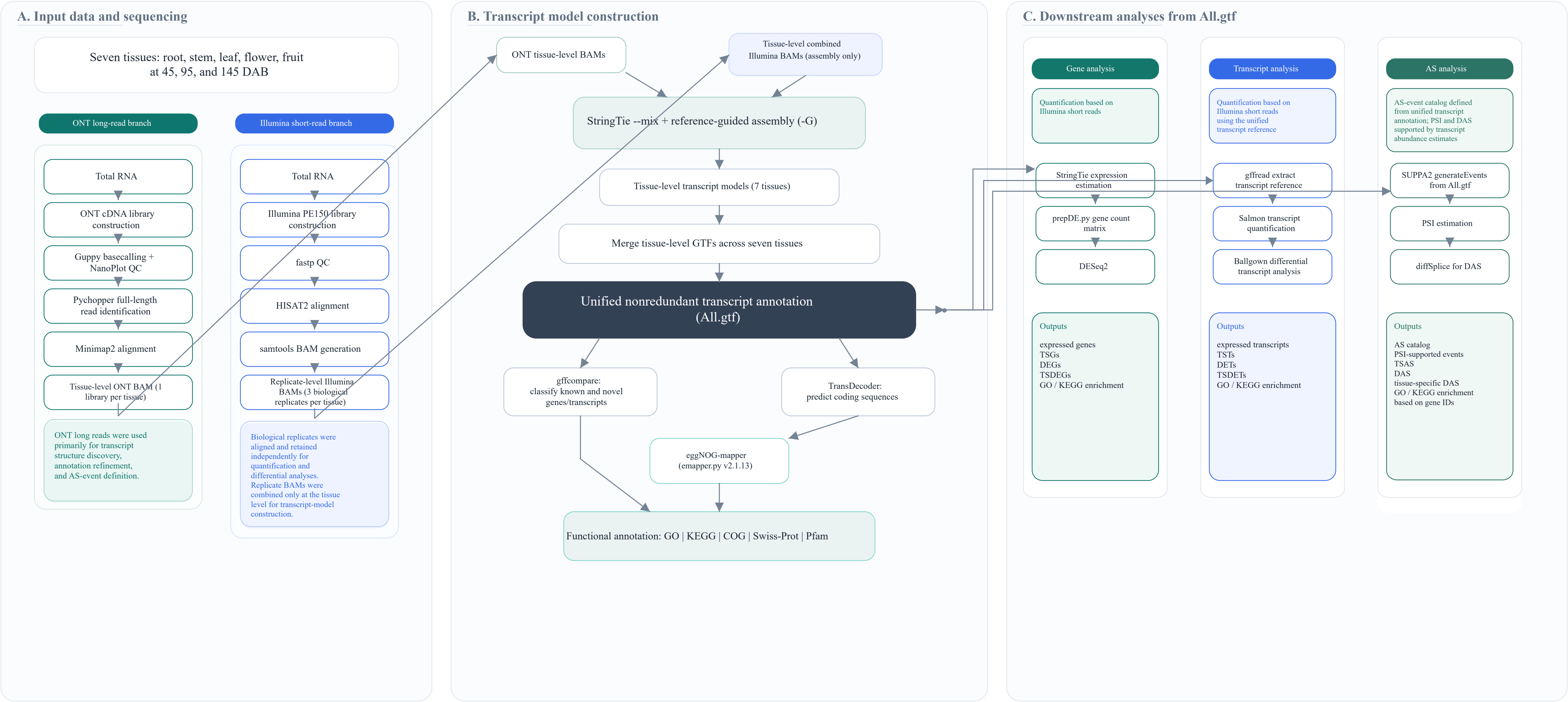

Corrected/final analysis framework used for manuscript revision: unified annotation (All.gtf) -> gene, transcript, and AS analyses with consistent downstream references.
